# Rejection Sensitivity Mediates the Relationship Between Social-Interpersonal Stressors and Depressive Symptoms in Military Context

**DOI:** 10.3389/fpsyt.2020.00447

**Published:** 2020-06-18

**Authors:** Jia Wang, Xiaotong Cheng, Ke Xu, Huimin Xu, Huizhong Wang, Zhengzhi Feng

**Affiliations:** ^1^Department of Developmental Psychology for Armyman, School of Psychology, Army Military Medical University, Chongqing, China; ^2^Graduate School, Army Military Medical University, Chongqing, China

**Keywords:** social-interpersonal stressors, rejection sensitivity, depressive symptoms, military, mechanism

## Abstract

**Background:**

Depression is pervasive in the military context and is likely to elicit lasting negative effects on health. Based on interpersonal models, social-interpersonal stressors are significantly associated with the development and maintenance of depression. However, little is known about the mechanisms by which these stressors increase the risk of depression in terms of social relationships. Rejection sensitivity, which refers to people who are sensitive to social rejection and tend to anxiously expect, readily perceive, and overreact to it, may play an underlying role in this process, as it is formed through social-interpersonal stressors and then aggravates further symptoms of depression.

**Objectives:**

The current study aimed to examine the mediating effects on the relationship between social-interpersonal stressors and depressive symptoms in the military context.

**Methods:**

This study recruited 600 soldiers aged from 17 to 36 (M = 21.80; SD = 2.99; 100% males) with a cluster sampling method who completed Social-Interpersonal Stressors subscale, Rejection Sensitivity Questionnaire (RSQ), and Self-Rating Depression Scale (SDS). Mediation analyses examined the underlying mechanism between social-interpersonal stressors and depressive symptoms.

**Results:**

The results support the hypothesis and indicate that rejection sensitivity mediates the association between social-interpersonal stressors and depressive symptoms (*B* indirect = 0.02, *p* < 0.001, 95% *CI*= 0.005 to 0.044).

**Conclusions:**

The findings suggest that interventions designed to desensitize individuals' high levels of rejection sensitivity may help to decrease their risk of depressive symptoms in the military environment. Rejection sensitivity is an important mechanism underpinning the development of depressive symptoms. Other theoretical and applied implications for prevention of depressive symptoms in the military context are discussed.

## Introduction

Depression is an important public health problem that has the greatest negative effect on health (1); the worldwide prevalence of MDD (major depressive disorder) was 3% during the period from 1990 to 2015 (2). Additionally, depression is associated with increased suicide rates, decreased quality of life, and poor social functioning (3). Specifically, in the military context, the impacts of depression can be exacerbated since military populations encounter additional special stressors, which are associated with the high risk of soldiers' mental health deterioration (4), including potential threat or danger, combat exposure, geographical separation, strict hierarchy, conflict between ranks and limited communication with families (5, 6). A meta-analysis found the evidence that combat experience substantially increases the risk of depression (*OR* = 1.60, 95%, *CI* = 1.09–2.35) (7). Further, there are a number of studies on the higher prevalence of major depression disorders and symptoms in military settings. For example, Gadermann et al. used 25 epidemiological studies to estimate the prevalence of DSM-IV major depression in U.S. military personnel; the results indicated prevalence rates of 12.0% among those currently deployed, 13.1% among those previously deployed and 5.7% among those never deployed (8). As is revealed by Warne et al., 15.9% endorsed moderate or more severe current depressive symptoms in U.S. entry-level training soldiers (9). In addition, the prevalence of major depression in Australian Gulf War veterans 20 years after the war was 9.7% among 697 Gulf War veterans, which was slightly more severe than a military comparison group (7.7%) (10). According to the 2016 Survey of Mental Disorders in the Republic of Korea, the prevalence of major depressive disorder was 5.0%, which was higher than the global prevalence of depression (3%), and 8.6% military officers had depressive symptoms among a sampling of 2047 participants (11). In China, Feng et al. studied 14,000 participants and showed that the prevalence of depressive symptoms in Chinese army men is 18.1% (12). Interestingly, the incidence varies with deployment exposure as well as operational missions; the prevalence of depressive symptoms in Chinese soldiers ranged from 5.20% to 51.56% (13, 14). Taken together, there is substantial evidence that military stressors increase the risk of depressive symptoms. Therefore, it is essential to reveal the underlying processes related to depressive symptoms to develop effective clinical strategies, especially in the military context, in which the incidence rates of depressive related disorders and symptoms are significantly higher than in other contexts (15).

According to interpersonal models of depression, social-interpersonal stressors are crucial risk factors concerning the etiology and course of depression (16–18). Rudlph's (19) research clearly showed that interpersonal stressors are associated with depressive symptoms and that interpersonal processes are involved in the development and perpetuation of depression. In the military settings, tremendous social-interpersonal stressors could be confronted by soldiers due to their specific environment, such as deployments or operational missions, unfamiliar cultural environment, isolated physical environment, and separation from and communication restrictions with family members (20). Nonetheless, troops are considered as a highly disciplined unit in which soldiers always share living quarters for significant missions or duties during service periods and separate from family members (21). Maintaining these close social bonds with comrades and leaders is essential for organizational morale and effectiveness (22). Furthermore, strains among peers, leaders, and family members, and a lack of support from them, may accelerate depressive symptoms (23–25).

From the perspective of well-established diathesis–stress theories, depression is a result of the interaction between vulnerability or predisposition (diathesis) and life stress (26). On the one hand, numerous social-interpersonal stressors have a long-lasting adverse effect on mental health, especially depression in military settings (7). On the other hand, for depressive individuals, their vulnerability or predisposition and being confronted with social-interpersonal stressors simultaneously account for their depression (26). One of vital predispositions may be rejection sensitivity, in which people who are sensitive to social rejection tend to anxiously expect, readily perceive, and overreact to such rejection (27). Humans tend to develop a sense of belonging to others rather than being rejected or isolated (28). Regarding military personnel under physically restricted and highly concentrated circumstances, to strengthen interpersonal relationships, social connections, and emotional support, avoiding rejection by others, are indispensable (20). Based on attachment theory, when foster parents tend to react to children's needs with rejection or neglect, children will develop insecure working models that are full of anxieties (29). In other words, individuals who suffer from social-interpersonal stressors (30, 31), especially those rejected by others, experience a high level of rejection sensitivity (27).

Rejection sensitivity is the core feature of atypical depression (3). Many empirical studies have demonstrated that rejection sensitivity is a risk factor for the etiology of depression (32–34). A meta-analysis review of 43 studies showed that there is a signiﬁcant association between rejection sensitivity and depression (pooled *r* = 0.332; *p* < 0.001) (35).

Previous studies have investigated the underlying cognitive and neural mechanisms by which social-interpersonal stressors can influence depression. Once individuals experience social defeat or rejection, those who feel a high level of social stress induce a cognitive change that may include negative self-referential cognitions (27) and elicit biological changes that may evoke depression, including prolonged and higher levels of cortisol reactivity (36), inflammatory responses (37), and dysregulation of the MAX-MYC network in the brain (38). When taking the individuals' the hyper-sensitivity to rejection predisposition into consideration, social-interpersonal stressors may activate differential effects on cognitive pathways and brain regions involved in processing social-interpersonal stress.

Thus, to protect military personnel from depression stemming from overwhelming social-interpersonal stressors, the possible mechanisms related to such depression need to be understood. Although significant relationships among social-interpersonal stressors, rejection sensitivity, and depression have been shown by numerous empirical studies, few of them have directly examined the mediating mechanism. The evaluation of depressive symptoms and their likely severity in military settings and identification of the underlying mechanisms are essential requirements for developing a comprehensive understanding of military depressive symptoms and for developing interventions based on social-interpersonal stress-rejection sensitivity for promoting soldiers' mental health and for enhancing unit fitness. Thus, our hypotheses are that the prevalence of depressive symptoms in our cohort was at a higher level in the military context, and rejection sensitivity mediates the association between relational stressors and depressive symptoms.

## Materials and Methods

### Participants

The participants were 600 soldiers recruited from armed forces with cluster sampling. The study was approved by the ethics committee of Army Medical University. Participants gave written informed consent before completing the questionnaires. Participation was voluntary and anonymous. Participants had a mean age of 21.80 ± 2.99 years; all of them were male; the mean length of military service was 3.46 ± 2.55 years; 566 participants were single (94.40%), and 34 were married (5.70%); and 64 participants had a low education level (10.70%), 425 had a moderate education level (70.80%), and 111 had a high education level (18.50%).

### Research Measures

#### Social-Interpersonal Stressors

The six-item social-interpersonal stressor subscale was chosen to assess the extent to which soldiers were confronted with social stress from leaders, peers, and lovers (39) (e.g., “The relationships with my fellow soldiers were strained. I will contradict my superiors.”). In the present study, Cronbach's α coefficient was 0.80.

#### Rejection Sensitivity

Rejection sensitivity was assessed with the Chinese version of the Rejection Sensitivity Questionnaire (RSQ) based on Downey's rejection sensitivity questionnaire (40). The RSQ consists of 18 interpersonal situations in which rejection is possible. Answers to the hypothetical situations varied along two dimensions: (a) the degree of anxiety and concern about the outcome and (b) expectations of acceptance or rejection. Anxiety and expectation were both rated on a 6-point Likert scale, with anxiety rated from 1 (not at all anxious) to 6 (very anxious) and expectation rated from 1 (very unlikely) to 6 (very likely). The rejection sensitivity score was calculated by multiplying the score for the degree of anxiety by the expectation of rejection (expectation of rejection = 7 - expectation of acceptance), and a total score was computed by summing the rejection sensitivity scores for each situation. Internal consistency was α=0.80), and test-retest reliability was *r* = 0.89. In the Chinese version of the RSQ, confirmatory factor analysis indicated that there were three factors involved, namely, leaders' rejection sensitivity, peers' rejection sensitivity, and lovers' rejection sensitivity. The three-factor model fit indices of the Chinese version of the RSQ in the military population were perfect (41). In the current sample, Cronbach's α coefficient was 0.84, indicating that reliability and validity were good.

#### Depressive Symptoms

Depressive symptom was measured using the self-rating depression scale of Zung (42), reflecting four groups of symptoms of depressive states: pervasive psychic disturbance, physiological disturbance, psychomotor disturbance, and psychological disturbance. The scale consists of 20 items, rated 1 (none or a little of the time) to 4 (most or all of the time), ranging from 20 to 80. Speciﬁcally, the scores of the 20 items were added and multiplied by 1.25 to convert them into standard scores. According to the norm for Chinese soldiers (43), scores ranging from 53 to 62 reﬂect mild depressive symptoms, scores ranging from 63 to 72 reflect moderate depressive symptoms and scores above 72 reflect major depressive symptoms. In the present sample, the scores had high internal consistency (α=0.80).

### Procedure

The survey was guided by a professional psychology researcher. The survey procedure was standard. Participates were asked whether they wanted to complete the survey. Only participants who completed the whole questionnaire were included in the sample. Participants with missing or irregular answers were excluded.

### Statistical Analysis

The descriptive statistics and correlation analyses of the outcome measures were performed using SPSS19.0. The mediation model mentioned in the introduction was tested with structural equation modeling by AMOS 19.0.

All analyses were conducted using SPSS19.0 and AMOS19.0.

The present status of depressive symptoms in soldiers was first tested with ANOVA and *t* test. Then, Pearson bivariate correlations were calculated to identify intervariable correlations. A simple mediation effect was examined using structural equation modeling.

## Results

### Overall Situations of Depressive Symptoms

According to the depression norm for Chinese soldiers, the prevalence of depressive symptoms in our military cohort was 28.4%: 148 participants had mild depressive symptoms (24.7%), 22 participants had moderate depressive symptoms (3.6%), and 1 participant had major depressive symptoms (0.2%). In terms of educational factors, the depressive symptoms score for low education level individuals' was significantly higher than that for high education level, *F* (2,597) = 5.25, *p* < 0.01. There was no difference in depression level between single and married individuals. To further explore demographic variables and risk factor differences between the group with depressive symptoms (*n* = 171) and the group with non-depressive symptoms (*n* = 429), results indicated that there were no differences between these two groups in age (*t* (598) = −0.08, *p* > 0.05) or the length of military service (t (598) = 0.04, p>0.05). Moreover, the scores for social-interpersonal stressors (*t* (598) = 5.69, *p* < 0.01) and rejection sensitivity (*t* (598) = 2.56, *p* < 0.05) in the depressive symptoms group were significantly higher than those in the non-depressive symptoms group (**Table 1**).

**Table 1:** Test of the variable differences between the depressive symptoms and non-depressive symptoms groups.

	Depressive symptoms group (N = 171)	Non-depressive symptoms group (N = 429)	*T*
	M ± SD	M ± SD	
1. Age	21.77 ± 2.91	21.79 ± 3.02	−0.08
2. Length of military service	3.45 ± 2.59	3.44 ± 2.54	0.04
3. Social-interpersonal stressors	15.76 ± 3.91	13.83 ± 3.68	5.69**
4. Rejection sensitivity	8.76 ± 2.93	8.13 ± 2.66	2.56**

M, means; SD, standard deviations.

** p < 0.01.

### Correlations Among Social-interpersonal Stressors, Rejection Sensitivity, and Depressive Symptoms

A simple correlation analysis revealed that social-interpersonal stressors were positively associated with rejection sensitivity (*r*=0.20, *p* < 0.01) and depressive symptoms (*r*=0.31, *p* < 0.01) and that rejection sensitivity was positively correlated with depressive symptoms (*r*=0.16, *p* < 0.01) (**Table 2**).

**Table 2:** Means (M), standard deviations (SD), and correlations for all variables.

	M ± SD	1	2
1. Social-interpersonal stressors	14.38 ± 3.84		
2. Rejection sensitivity	8.38 ± 2.80	0.20**	
3. Depressive symptoms	39.08 ± 7.41	0.31**	0.16**

** p < 0.01.

### Rejection Sensitivity as a Mediator of the Relationship Between Social-Interpersonal Stressors and Depressive Symptoms

**Figure 1:**
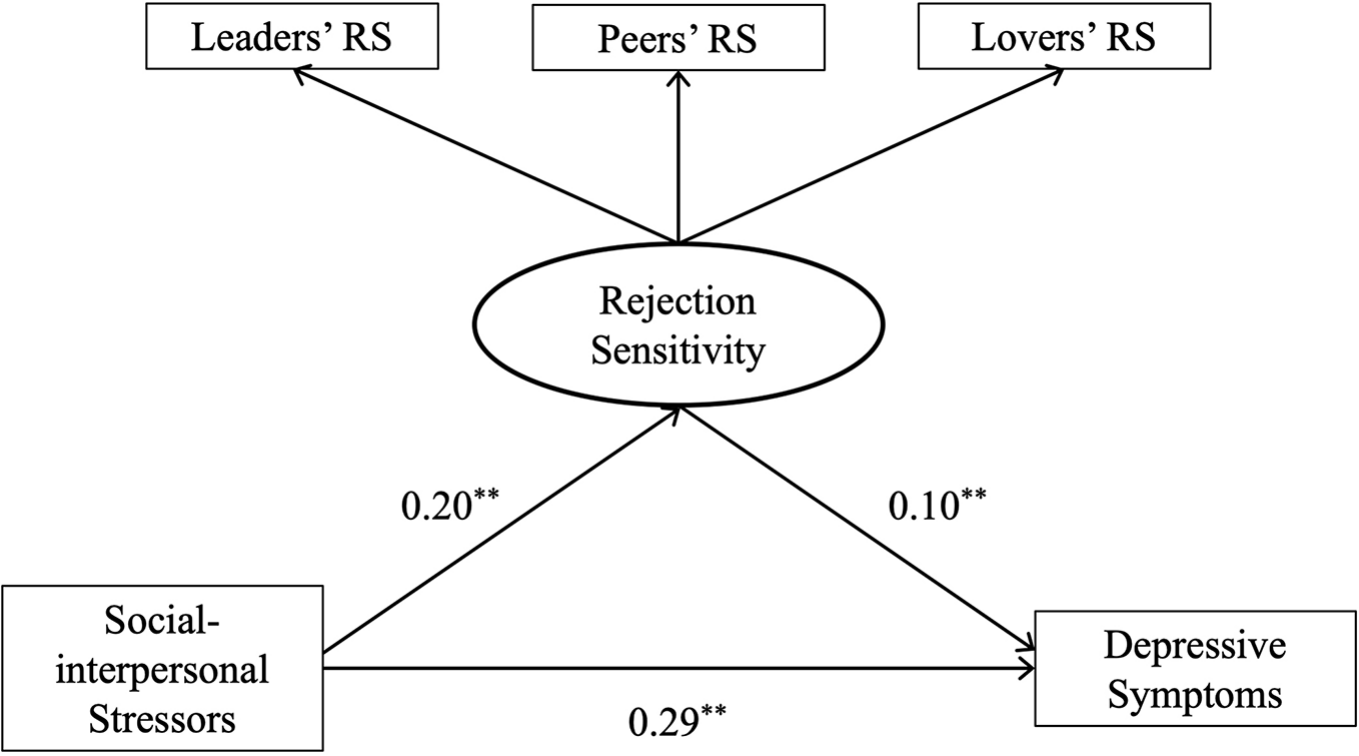
Rejection sensitivity as a mediator through which social-interpersonal stressors increase depressive symptoms. **p < 0.01; RS, rejection sensitivity.

In the mediation analysis, social-interpersonal stressors were entered as independent variables, depressive symptoms as the dependent variable and rejection sensitivity as the mediator. **Table 3** shows that the relationship between social-interpersonal stressors and depressive symptoms is partially mediated by rejection sensitivity (*B*_indirect_= 0.02, *p* < 0.001, *CI* = 0.005 to 0.044). The results of structural equation modeling showed that the overall model yielded a satisfactory fit, CMIN/DF = 1.374, *p*= 0.24>0.05, GFI = 0.996, AGFI = 0.986, NFI = 0.991, IFI = 0.997, CFI = 0.997, and RMSEA = 0.025. The model explained 10.70% of the variance in depressive symptoms (*F* (2,597) = 35.74, *p* < 0.001) (**Figure 1**).

**Table 3:** Test of the mediation model.

IV	DV		Coeff.	SE	95%CI	
					LL	UL
Social-interpersonal stressors	Depressive symptoms	Total effect	0.31	0.034		
		Direct effect	0.29	0.044		
		Indirect effect	0.02	0.009	0.005	0.044

IV, independent variable; DV, dependent variable; CI, confidence interval; LL, lower level; UL, upper level.

## Discussion

The present study evaluated the hypothesis that rejection sensitivity mediates the relationship between social-interpersonal stressors and depressive symptoms. Before a mediation model could be established, soldiers' depressive symptom situations had to be analyzed.

Our study found that the prevalence of soldiers' depressive symptoms in our cohort was 28.4%, which is higher than the norm, though it was within range of the ratio among Chinese soldiers (14). This finding could be related to the different severities of military deployment exposure in which the adverse effects on mental health were observed (4, 8). Moreover, Zung, who developed the Zung Self-Rating Depression Scale (SDS), proposed that the norm of SDS among the Chinese was higher than that from the United States due to cultural diversity (42). Importantly, our results indicated that low education level was a risk factor for more depressive symptoms, consistent with a previous study that determined that a higher education level was related to the capability for psychological adjustment (7, 8). There were no significant relationships found in this study sample when marital status, age, and the length of military service were examined as risk factors for depressive symptoms. Compared with the non-depressive symptoms group, the scores for social-interpersonal stressors and rejection sensitivity were significantly higher. Thus, the results certify the viewpoint that depressed military personnel have a markedly elevated level of social-interpersonal stress and rejection sensitivity when they are in more vulnerable circumstances.

Our results confirmed that social-interpersonal stressors have a direct adverse effect on depression symptoms, which is consistent with previous studies. Military personnel are exposed to a great deal of social-interpersonal stressors. Their relationships with leaders, peers, and lovers play a critical role regarding these stressors since soldiers always share living quarters for significant missions or duties during their service period (20, 21). Similarly, in the present study, soldiers' mean age was 21.80 ± 2.99 years. Regarding life developmental stages, approximately 20 to 40 years of age is considered early adulthood, in which individuals need to resolve their “intimacy vs. isolation” life crises. In other words, they need to develop the capacity for closeness and commitment to others, or otherwise, they will become isolated and alone and will have inability to connect to others in psychologically meaningful ways (44). For young army men, interpersonal relationships are one of the critical skills required for soldiers in the future (45). However, the prevalence of interpersonal violence among soldiers is approximately 16%, and such violence is significantly associated with depression (46).

In military settings, three types of intimate relationships are common: relations with leaders, peers, and lovers. First, to maintain mutual social interactions with leaders, this kind of adaptive relation is based on military leadership in alleviating depression (47) since a strain with the leader will lead to soldiers having less support available to them, increasing social problems and damaging organizational morale and effectiveness, which may undermine mental health (22–24), including depressive symptoms (25, 48, 49). Additionally, the development of close social ties with peers is helpful in enhancing the sense of belonging and expanding the social networks or social support systems of soldiers; through this protective effect, these bonds with peers buffer depression and improve the mental health of soldiers as well as unit morale (50–54). Finally, a meta-analysis highlighted that, compared with other marital statuses, being married is a protective factor against depression (55) because family function and social support from family are negatively associated with depression in soldiers (56).

Based on previous research, the current study proposed that stressors from leaders, peers, and lovers might elicit depression in soldiers to the extent that rejection sensitivity is increased. These results align with our hypothesis.

According to Beck's cognitive model of depression, once stressful events occur, biased attention, thoughts, rumination, memory, and dysfunctional schemas are consistently linked with the onset and maintenance of depression (57). Social rejection stressors are significantly associated with rejection sensitivity (58, 59). The neural dynamics of the rejection sensitivity study showed that under rejection cues, individuals with high rejection sensitivity have decreased activity in the left lateral PFC regions related to cognitive control (60). Additionally, individuals with high rejection sensitivity pay attention to potential rejection cues in a biased manner (61), have schema-congruent information processing biases (62), experience much more rumination (63), and have interpretation biases (64). Consequently, through the distortion effect of rejection sensitivity, people with a high level of social stress experience more depressive symptoms than do those with a low level of social stress (32, 65, 66).

Social stress evokes the anterior cingulate cortex, which overlaps circuitry with physical pain. This neural alarm system is evolutionarily adaptive for humans (67). When individuals with major depression are confronted with social pain, the rejection sensitivity level would deteriorate, activating the greater amygdala, insula, anterior cingulated cortex in these individuals (68–70). In individuals with a high level of rejection sensitivity, rejection stressors could automatically activate the defensive motivational system (71). This self-protective system tends to make individuals flee or escape from social stress (72), thus helping to predict more depressive symptoms (73, 74).

## Limitations and Conclusions

The current study has several limitations. First, data were collected in a sample of nonclinical and predominantly male army recruits. Therefore, the generalization of the findings is limited. In addition, a cross-sectional method was used. Further studies should consider these limitations and focus on the replication of the findings in a prospective longitudinal design with clinical samples.

In military settings, the prevalence of depressive-related disorders or symptoms could be assessed at a higher level than in normal populations. Therefore, utilizing validated measures for the early detection of depression, coupled with psychological education, psychological counseling and therapy, and referral to hospitals for antidepressant medication treatment when necessary to maintain the mental fitness of soldiers is imperative. Crucially, morale and unit effectiveness are always emphasized as the priority consideration in the military context. From the perspective of social relationships, determining potential risk factors could prevent episodes of depression. For the individuals who at high rejection sensitivity when encountering social-interpersonal stressors were susceptible to depression. Thus, the metacognition based on psychological education and training should be implemented to intervene, which mainly about how to detect the rejection cues in social interaction with desensitization way and how to self-regulate the biased information and negative emotions related with rejection. This is the first study to assess the relationships among social-interpersonal stressors, rejection sensitivity and depressive symptoms, and the results showed a partial mediating effect of rejection sensitivity on the association between social-interpersonal stressors and depressive symptoms. Interventions aimed at decreasing social-interpersonal stressors therefore need to consider rejection sensitivity. Moreover, identifying the risk factors for rejection sensitivity and social stress is important since there is accumulating evidence indicating that both factors have a pernicious effect on depressive symptoms.

## Data Availability Statement

The raw data supporting the conclusions of this article will be made available by the authors, without undue reservation.

## Ethics Statement

The studies involving human participants were reviewed and approved by Ethics committee of the army medical university. The patients/participants provided their written informed consent to participate in this study. Written informed consent was obtained from the individual(s) for the publication of any potentially identifiable images or data included in this article.

## Author Contributions

JW, XC, and ZF contributed to the design and conception of this study. All authors actively took part in the process. KX, HX, and HW planned and participated in the statistical analysis. JW, XC, and ZF participated in a critical review of the manuscript. All authors read and approved the final manuscript.

## Funding

This study was financially supported by Training Program of the Social Science Planning of Chongqing (No.2016PY51) and Natural Science and foreword exploratory Foundation of Chongqing (No. cstc2018jcyjAX0276).

## Conflict of Interest

The authors declare that the research was conducted in the absence of any commercial or financial relationships that could be construed as a potential conflict of interest.
